# Desflurane combined with ciprofol or esketamine improves postoperative symptoms without compromising early neurocognitive recovery after intracranial aneurysm endovascular coiling

**DOI:** 10.3389/fmed.2026.1829717

**Published:** 2026-05-29

**Authors:** Tenghuan Wang, Na Xing, Huixin Li, Yuanyuan Mao, Dan Cheng, Yanan He, Sheng Guan

**Affiliations:** 1 Department of Anesthesiology, Pain and Perioperative Medicine, The First Affiliated Hospital of Zhengzhou University, Zhengzhou, China; 2 Department of Imaging and Interventional Medicine, The First Affiliated Hospital of Zhengzhou University, Zhengzhou, China

**Keywords:** ciprofol, desflurane, endovascular coiling, esketamine, intracranial aneurysm, neurocognitive recovery, perioperative adverse events

## Abstract

**Objective:**

This randomized controlled trial aimed to compare the effects of three anesthetic regimens—desflurane + 0.9% saline (DS), desflurane + ciprofol (DC), and desflurane + esketamine (DE)—on early postoperative neurocognitive recovery and perioperative adverse events in patients undergoing endovascular coiling for intracranial aneurysms.

**Methods:**

This randomized controlled trial enrolled 210 patients scheduled for urgent endovascular coiling of intracranial aneurysms. Patients were randomly assigned in a 1:1:1 ratio to receive DS, DC, or DE. Neurocognitive function was evaluated using the Montreal Cognitive Assessment (MoCA) before surgery, on postoperative day 1 (POD 1), and on postoperative day 5 (POD 5). Secondary outcomes included the incidence of postoperative nausea and vomiting (PONV), dizziness, numerical rating scale (NRS) pain scores, and the requirement for rescue antiemetic medication. This study was registered at the Chinese Clinical Trial Registry (ChiCTR2500097866).

**Results:**

The MoCA scores improved significantly over time across all groups (*p* < 0.001), with no significant differences in neurocognitive recovery trajectories among groups (interaction effect, *p* = 0.196; group effect, *p* = 0.729). The incidence of PONV differed significantly: 10.3% in the DC group, 17.9% in the DE group, and 44.9% in the DS group (*p* < 0.001). The DC group also exhibited the lowest incidence of dizziness (8.8%) and the lowest metoclopramide requirement (*p* = 0.012). Postoperative pain scores were significantly lower in the DE group than in the DC group (adjusted *p* = 0.026).

**Conclusion:**

In patients undergoing endovascular coiling for intracranial aneurysms, desflurane-based anesthesia supplemented with ciprofol or esketamine does not compromise early neurocognitive recovery. Desflurane plus ciprofol effectively reduces PONV and dizziness, whereas desflurane plus esketamine provides superior analgesia. These findings support personalized, symptom-targeted anesthetic strategies to enhance perioperative safety and recovery.

## 1 Introduction

Aneurysmal subarachnoid hemorrhage (aSAH) remains a significant global public health challenge (1, 2). Endovascular coiling is a widely used treatment for intracranial aneurysms (3, 4), but postoperative neurocognitive dysfunction, a common complication, can severely impair patients’ long-term quality of life (5, 6). Perioperative anesthetic management during these procedures is critical, requiring meticulous attention to hemodynamic stability (7), physiological homeostasis (8), rapid emergence, and the prevention of postoperative nausea and vomiting (PONV). Anesthetic agents with potential neuroprotective properties or the ability to alleviate procedure-related neurological injury, therefore, deserve special consideration.

Emerging evidence suggests that desflurane may offer neuroprotective benefits, including reduced cerebral infarct volume, the attenuation of vasospasm, and improved neurological outcomes (9–11). Its low blood–gas solubility also facilitates rapid postoperative recovery (12–14). Nevertheless, desflurane is associated with higher rates of PONV and dizziness compared with intravenous anesthetics, along with concerns regarding cost and environmental impact (15). These limitations highlight the need for its prudent use and the exploration of optimized anesthetic regimens.

Intravenous anesthetics, such as ciprofol and esketamine, possess distinct pharmacodynamic advantages that make them promising adjuvants in neuroanesthesia. However, their impact on neurocognitive recovery following intracranial aneurysm coiling remains insufficiently characterized. Although previous studies have investigated the influence of anesthesia on neurocognition and postoperative outcomes (16–19), direct comparisons between desflurane monotherapy and its combinations with ciprofol or esketamine in this specific surgical setting are lacking. Conducting rigorous comparative research is, therefore, clinically imperative. Such an investigation is essential to inform tailored perioperative strategies and establish evidence-based pathways for enhancing neurocognitive recovery. Given the unique demands of neurointerventional surgery and the potential synergistic effects of multimodal anesthesia, further studies evaluating neurocognitive performance and recovery metrics are vital to advance precision anesthesia practice and improve patient care in neurointerventions.

We hypothesized that adding ciprofol or esketamine to desflurane anesthesia may lead to improved early postoperative neurocognitive recovery relative to desflurane monotherapy in patients undergoing endovascular coiling for aSAH.

## 2 Methods

This trial was registered with the Chinese Clinical Trial Registry (ChiCTR; URL: https://www.chictr.org.cn/; Registration No.: ChiCTR2500097866). The study was conducted in accordance with the Declaration of Helsinki and approved by the Ethics Committee of the First Affiliated Hospital of Zhengzhou University (Approval No.: 2024-KY-2205-002). Written informed consent was obtained from all participants before study enrollment. The study was performed and reported in compliance with the Consolidated Standards of Reporting Trials (CONSORT) guidelines.

### 2.1 Subjects

This prospective, randomized controlled trial consecutively enrolled patients aged 20–80 years, with an American Society of Anesthesiologists (ASA) physical status of I–III, who underwent urgent endovascular coiling for aneurysmal subarachnoid hemorrhage (aSAH) with an admission Hunt-Hess grade of I–III. Recruitment was performed at the First Affiliated Hospital of Zhengzhou University between September 2024 and March 2025.

The exclusion criteria were as follows:

(1) A confirmed history of psychiatric disorders, including schizophrenia, bipolar disorder, or major depressive disorder.
(2) An inability to complete the baseline Montreal Cognitive Assessment (MoCA) preoperatively due to impaired consciousness or agitation.
(3) The regular use of sedative-hypnotic or anxiolytic medications for more than 4 weeks before surgery.
(4) Participation in another interventional clinical trial within 3 months before enrollment.
(5) Any other condition judged by the investigator to compromise reliable outcome assessment or patient safety, such as an expected survival of <72 h.

### 2.2 Randomization and blinding

An independent research coordinator performed stratification according to sex and age (20–80 years, divided into 10-year intervals). Within each stratum, patients were randomly assigned to the three study groups in a 1:1:1 ratio using a computer-generated randomization sequence with a fixed block size of six, applied in the order of enrollment.

Allocation concealment was guaranteed by a study nurse who was not involved in patient care. This nurse generated, stored, and implemented the randomization list, and prepared all study medications (or matching placebo) in identical opaque syringes labeled only with the patient’s unique study identification number.

Patients, attending anesthesiologists, surgeons, and all outcome assessors were blinded to the group allocation throughout the study. One blinded anesthesiologist administered anesthesia and recorded intraoperative and postanesthesia care unit (PACU) data. All operations were performed by a single surgeon who was blinded to the group assignment. A second blinded anesthesiologist collected the baseline data, and a third blinded anesthesiologist performed all postoperative neurological and cognitive assessments on postoperative day (POD) 1 and POD 5. This design ensured that evaluators had no access to intraoperative or group allocation data, thus maintaining blinding integrity.

For intraoperative drug-related adverse events (e.g., hypotension, bradycardia, and hallucinations), the attending anesthesiologist provided standardized symptomatic treatment per the study protocol without unblinding. In cases of severe, life-threatening adverse events (e.g., refractory hypotension and severe respiratory depression), unblinding could be requested to access group allocation, with detailed documentation in the case report form (CRF) (including unblinding time, reasons, and treatment measures). After unblinding, patients continued follow-up in accordance with the original protocol, and unblinded cases were independently analyzed in sensitivity analyses to assess their impact on the study results.

### 2.3 Anesthesia protocol

Upon arrival in the operating room, standard monitoring was applied, including electrocardiography (ECG), noninvasive blood pressure (NIBP) measured at the right brachial artery, and pulse oximetry (SpO_2_). Venous access was established in the left upper extremity.

Anesthesia was induced intravenously with etomidate (2 mg/kg), alfentanil (20 μg/kg), and cisatracurium (0.1 mg/kg). Following the loss of consciousness, an Ambu® AuraGain™ is manufactured by Ambu A/S (Ballerup, Denmark). Size #3 is indicated for patients weighing 30–50 kg, and Size #4 for 50–70 kg. But, based on our clinical experiencein Chinese populations, size 3# is suitable for female patients, while size 4# is suitable for male patients.^1^ Adjunctive medications administered at induction included penehyclidine hydrochloride (20 μg/kg), palonosetron (0.25 mg) (20, 21), and betamethasone (8 mg) (20, 21), followed by a 15-min infusion of dexmedetomidine (0.4 μg/kg).

Anesthesia was maintained with inhaled desflurane (4%–6%) and a continuous infusion of remifentanil at 0.1–0.2 μg·(kg·min)^−1^. Cisatracurium (0.05 mg/kg) was administered as a bolus every 30 min. Study drug infusions were initiated for maintenance sedation. The bispectral index (BIS) was maintained between 40 and 60. Hemodynamic variables (heart rate and blood pressure) were maintained within 20% of baseline values, with vasoactive agents used as needed.

At the conclusion of surgery, flurbiprofen axetil (50 mg) was administered intravenously for analgesia. Anesthesia was discontinued, and the patients were considered awake upon eye opening in response to a verbal command. LMA extubation was performed when the patients met the following criteria: eye opening, cooperative movement, a tidal volume >5 mL/kg, and a respiratory rate between 10 and 30 breaths per min. Patients were discharged from the postanesthesia care unit (PACU) upon achieving a Steward recovery score of ≥4.

### 2.4 Intervention groups

Patients were randomized to one of three maintenance regimens. Study drug infusions were started after anesthesia induction and continued until the end of surgery:

(1) Desflurane–0.9% saline group (DS group): 0.9% normal saline infused continuously at 0.08 mL·(kg·h)^−1^ combined with desflurane.
(2) Desflurane–ciprofol group (DC group): ciprofol (2.5 mg/mL) infused continuously at 0.08 mL·(kg·h)^−1^ combined with desflurane.
(3) Desflurane–esketamine group (DE group): esketamine (2.5 mg/mL) infused continuously at 0.08 mL·(kg·h)^−1^ combined with desflurane.

### 2.5 Data collection and outcome measures

Baseline demographic and clinical data were collected, including age, gender, weight, Hunt-Hess grade, comorbidities (hypertension, diabetes mellitus, and coronary heart disease), educational level, aneurysm location, and sleep quality as assessed by the Pittsburgh Sleep Quality Index (PSQI).

The primary outcome was the MoCA score evaluated at baseline (preoperatively), POD 1, and POD 5.

Secondary outcomes included the following:

(1) The incidence of postoperative nausea and vomiting (PONV) during the PACU stay, as assessed by patient report and nursing monitoring.
(2) The occurrence of postoperative delirium in the PACU, as evaluated using the Confusion Assessment Method for the Intensive Care Unit (CAM-ICU).
(3) Postoperative pain intensity in the PACU, as measured using the numerical rating scale (NRS, 0–10).
(4) Urinary catheter-related discomfort, as rated on a 4-point scale (0–3).

Other perioperative variables recorded included intraoperative and PACU hemodynamics (heart rate, blood pressure), BIS values, time to emergence (from the discontinuation of anesthetics to eye-opening on command), operative duration, fluid balance, agitation episodes, and the use of vasoactive agents (dopamine, metaraminol).

### 2.6 Statistical analysis

The sample size was calculated *a priori* using G*Power software (version 3.1.9.7). Based on pilot data, a repeated-measures analysis of variance (ANOVA) with between–within factors was used as the primary statistical model. To detect a small effect size (partial *η*^2^ = 0.011) with 95% power (*β* = 0.05) at a two-sided *α* = 0.05, with three groups and three repeated measurements, an assumed correlation among repeated measures of 0.810, and a nonsphericity correction (*ε*) of 0.520, a minimum of 174 patients was required. To account for an estimated 20% dropout rate, the total sample size was set to 210, with 70 patients per group.

Statistical analysis was performed using IBM SPSS Statistics 22.0 (IBM Corp., Armonk, NY, USA). Two-sided tests were used at the significance level of *α* = 0.05 and with 95% confidence intervals. Categorical data were presented as *n* (%). Normally distributed continuous data were expressed as the mean ± standard deviation (SD), and non-normally distributed data were expressed as the median (interquartile range, IQR).

Baseline comparisons were performed using the Pearson χ^2^ test or Fisher’s exact test for categorical variables, a one-way ANOVA for normally distributed continuous variables, and the Kruskal–Wallis H test for non-normally distributed variables. Primary outcomes were analyzed using a repeated-measures ANOVA. Secondary outcomes were analyzed using a one-way ANOVA (normal distribution) or the Kruskal–Wallis H test (non-normal distribution).

## 3 Results

**Figure 1:**
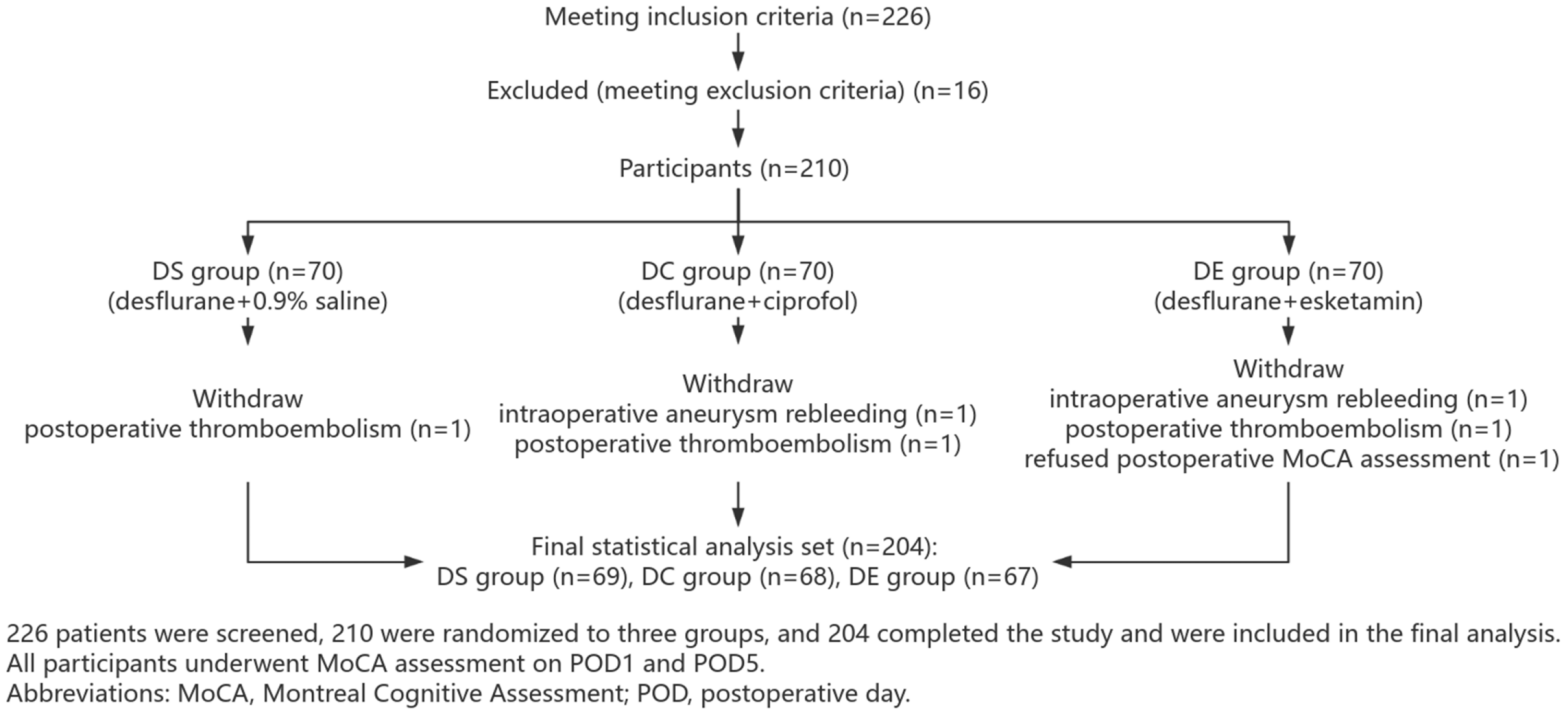
CONSORT flow diagram of patient enrollment, randomization, and follow-up.

Of the 210 patients initially enrolled, six were excluded from the final analysis due to intraoperative rebleeding (*n* = 2), postoperative thromboembolism (*n* = 3), or refusal to complete the postoperative cognitive assessment (*n* = 1) (Figure 1). The remaining 204 patients were included in the per-protocol analysis. Baseline demographic and clinical characteristics were balanced across the three intervention groups, with no statistically significant differences observed (Table 1).

**Table 1:** Baseline characteristics and intraoperative variables by study group.

Characteristic	DS (*n* = 69)	DC (*n* = 68)	DE (*n* = 67)	*p*-value
Demographics				
Age, mean ± SD, y	56.96 ± 9.07	56.09 ± 10.13	58.27 ± 10.90	0.449^‡^
Male sex, No. (%)	28 (40.6)	21 (30.9)	28 (41.8)	0.385^†^
BMI, mean ± SD, kg/m^2^	26.56 ± 14.57	25.29 ± 4.69	25.40 ± 3.79	0.667^‡^
Preoperative status				
Pre-SBP, mean ± SD, mmHg	135.16 ± 14.20	130.47 ± 15.75	134.33 ± 15.89	0.164^‡^
Pre-DBP, mean ± SD, mmHg	82.12 ± 9.12	80.88 ± 9.92	81.49 ± 9.35	0.745^‡^
Pre-PSQI score, mean ± SD	9.06 ± 1.15	9.12 ± 1.03	9.16 ± 1.10	0.851^‡^
Comorbidities, *n* (%)				
Hypertension	34 (49.3)	38 (55.9)	38 (56.7)	0.633^†^
Diabetes	9 (13.0)	9 (13.2)	10 (14.9)	0.941^†^
Coronary artery disease	6 (8.7)	7 (10.3)	10 (14.9)	0.492^†^
Smoking	6 (8.7)	8 (11.8)	6 (9.0)	0.800^†^
Motion sickness	25 (36.2)	23 (33.8)	20 (29.9)	0.728^†^
Aneurysm characteristics				
Hunt-hess grade I/II	28/41 (40.6/59.4)	22/46 (32.4/67.6)	22/45 (32.8/67.2)	0.528^†^
Internal carotid artery/Middle cerebral artery/anterior cerebral artery/vertebral and basilar arteries/others	28/16/2/10/13	33/15/4/10/6	27/9/3/12/16	0.411^†^
Intraoperative variables				
Fluid intake, mean ± SD, mL	878.99 ± 376.56	962.50 ± 427.19	862.69 ± 337.03	0.267^‡^
Fluid output, mean ± SD, mL	565.22 ± 346.34	529.85 ± 342.30	505.97 ± 299.56	0.575^‡^
Hemoglobin, mean ± SD, g/L	126.98 ± 12.38	127.53 ± 14.50	127.28 ± 11.15	0.968^‡^
Blood glucose, mean ± SD, mmol/L	6.23 ± 1.26	6.15 ± 1.40	5.97 ± 1.22	0.504^‡^
Time to emergence, mean ± SD, min	8.77 ± 1.67	8.87 ± 2.00	8.99 ± 1.71	0.792^‡^
SBP at emergence, mean ± SD, mmHg	139.67 ± 15.45	133.82 ± 14.00	138.22 ± 18.34	0.088^‡^
DBP at emergence, mean ± SD, mmHg	80.84 ± 11.78	79.90 ± 12.13	80.13 ± 14.24	0.903^‡^
HR at emergence, mean ± SD, bpm	73.32 ± 12.30	69.74 ± 12.19	71.78 ± 12.33	0.233^‡^
Duration of surgery, mean ± SD, min	66.28 ± 32.96	69.04 ± 28.26	62.34 ± 27.04	0.418^‡^
Metaraminol use, mean ± SD, μg·(kg·min)^−1^	0.98 ± 0.36	0.99 ± 0.35	0.98 ± 1.42	0.996^‡^
Dopamine use, mean ± SD, μg·(kg·min)^−1^	2.22 ± 0.67	2.25 ± 0.78	2.27 ± 3.55	0.986^‡^

^†^Data are reported as *n* (%), with *p*-values derived from Pearson’s chi-square test.

^‡^Data are reported as mean ± SD and analyzed using one-way analysis of variance (ANOVA).

BMI, body mass index; SBP, systolic blood pressure; DBP, diastolic blood pressure; HR, heart rate; DOS, duration of surgery; PSQI, Pittsburgh Sleep Quality Index; DS, desflurance–0.9% saline group; DC, desflurance–ciprofol group; DE, desflurance–esketamine group.

### 3.1 Neurocognitive recovery

A repeated-measures analysis of variance (ANOVA) demonstrated a significant main effect of time on Montreal Cognitive Assessment (MoCA) scores across all groups (F [1.19, 176.01] = 34.74, *p* < 0.001; partial η^2^ = 0.147). *Post hoc* testing revealed a significant and progressive increase in MoCA scores from the preoperative baseline (23.14 ± 0.27) to postoperative day 1 (mean difference = 0.72; 95% confidence interval [CI]: 0.24–1.12; *p* = 0.001) and to postoperative day 5 (mean difference = 1.31; 95% CI: 0.89–1.73; *p* < 0.001).

Critically, there was no significant group × time interaction (*p* = 0.196) nor a main effect of group (*p* = 0.729), indicating that the trajectory of early postoperative neurocognitive recovery was comparable among patients receiving desflurane monotherapy or desflurane combined with either adjuvant agent (Table 2). Furthermore, subgroup analyses stratified by age yielded consistent results, with similar temporal trends and comparable between-group differences observed across all age subgroups (Table 3).

**Table 2:** RM–ANOVA of MoCA scores by group and time.

Group	POD-1	POD1	POD5	*F* (df)	*p*	Particle *η*^2^	Post hoc (bonferroni)
DS	23.07 ± 3.74	23.80 ± 3.99	24.24 ± 3.92				POD-1 < POD1
DC	23.68 ± 3.78	23.97 ± 3.96	24.66 ± 3.68				POD1 < POD5
DE	22.66 ± 4.12	23.79 ± 4.33	24.43 ± 4.05				POD-1 < POD5
Time				34.74 (1.19, 176.01)	<0.001	0.147	
Time×group				1.61 (2.38, 16.36)	0.196	0.016	
Group				0.32 (1.19,26.51)	0.729	0.003	

Mauchly’s test of sphericity indicated a violation of the sphericity assumption (W = 0.317, *χ*^2^ = 229.48, *p* < 0.001); thus, the Greenhouse–Geisser correction was applied (*ε* = 0.594). Post hoc pairwise comparisons were adjusted using the Bonferroni method. Statistical significance was set at *α* = 0.05.

POD-1, operative day −1; POD1, postoperative day 1; POD5, postoperative day 5; DS, desflurance–0.9% saline group; DC, desflurance–ciprofol group; DE, desflurance–esketamine group.

**Table 3:** RM-ANOVA of MoCA scores in different age subgroups.

Subgroup	Category	Group	POD-1	POD1	POD5	Statistical effect	*F* (df)	*p*	Particle *η*^2^
Age (year)	20–64	DS	23.58 ± 3.68	24.40 ± 3.75	24.89 ± 3.68	Main effect of time	32.03 (1.25, 136.97)	<0.001	0.175
		DC	24.25 ± 3.46	24.57 ± 3.49	25.25 ± 3.23	Time×group interaction effect	1.09 (2.51, 9.36)	0.347	0.014
		DE	23.67 ± 3.90	24.76 ± 3.99	25.37 ± 3.61	Main effect of group	0.20 (2, 13.93)	0.822	0.003
	65–80	DS	21.07 ± 3.41	21.43 ± 4.11	21.71 ± 3.99	Main effect of time	4.26 (1.06, 33.53)	0.042	0.083
		DC	21.67 ± 4.30	21.87 ± 4.87	22.60 ± 4.48	Time×group interaction effect	0.64 (2.12, 10.12)	0.539	0.027
		DE	20.43 ± 3.76	21.67 ± 4.37	22.38 ± 4.30	Main effect of group	0.123 (2, 11.06)	0.885	0.005

Mauchly’s test of sphericity indicated a violation of the sphericity assumption; thus, the Greenhouse–Geisser correction was applied. Statistical significance was set at *α* = 0.05.

POD-1, operative day −1; POD1, post-operative day 1; POD5, postoperative day 5; DS, desflurance–0.9% saline group; DC, desflurance–ciprofol group; DE, desflurance–esketamine group.

### 3.2 Postoperative adverse events and symptomatology

The incidence of key perioperative adverse events differed significantly across the treatment groups (Table 4). The incidence of nausea was significantly higher in the desflurane–0.9% saline group (DS, 44.9%) than in the desflurane–ciprofol group (DC, 10.3%) and the desflurane–esketamine group (DE, 17.9%) (*p* < 0.001). Although the incidence of vomiting trended higher in the DS group, this difference did not reach statistical significance (*p* = 0.056). The incidence of dizziness varied significantly by group (*p* < 0.001), with the DC group exhibiting the lowest rate (8.8%). Regarding analgesic efficacy, postoperative pain scores were significantly lower in the DE group compared with the DC group (adjusted *p* = 0.026). In addition, the DC group also demonstrated the lowest requirement for rescue medication with metoclopramide (4.4%), which was significantly lower compared with other groups (*p* = 0.012), while no significant intergroup differences were observed in the incidence of agitation, delirium, or urinary catheter-related irritation (all *p* > 0.05). For hemodynamic parameters at PACU discharge, systolic blood pressure (SBP) differed significantly among groups (*p* = 0.012), with the DC group displaying significantly lower SBP values than the DE group (adjusted *p* = 0.003). No significant between-group differences were detected in diastolic blood pressure or intraoperative oxygen saturation (SpO_2_) (all *p* > 0.05).

**Table 4:** Postoperative adverse events and vital signs by group.

Outcome	DS (*n* = 69)	DC (*n* = 68)	DE (*n* = 67)	*p*	Post hoc analysis (bonferroni)
Adverse events					
Nausea, *n* (%)	31 (44.9%)	7 (10.3%)	12 (17.9%)	<0.001^†^	GS > GC, GS > GE
Vomiting, *n* (%)	12 (17.4%)	5 (7.4%)	4 (6.0%)	0.056^†^	—
Dizziness, *n* (%)	24 (34.8%)	6 (8.8%)	20 (29.9%)	0.001^†^	GS > GC, GE > GC
Agitation, *n* (%)	4 (5.8%)	2 (2.9%)	2 (3.0%)	0.615^†^	—
Delirium, *n* (%)	6 (8.7%)	1 (1.5%)	2 (3.0%)	0.094^†^	—
Catheter Irritation, median (IQR)	1 (1–1)	1 (1–1)	1 (1–1)	0.421^§^	—
Metoclopramide use, *n* (%)	15 (21.7%)	3 (4.4%)	10 (14.9%)	0.012^†^	GS > GC
Vital signs					
PACU–SpO_2_ (%), mean ± SD	97.5 ± 2.0	97.8 ± 1.8	95.8 ± 12.0	0.213^‡^	—
PACU–SBP, median (IQR)	135 (125–152)	133 (124–144)	144 (130–155)	0.012^§^	GE > GC
PACU–DBP, mean ± SD	84.1 ± 12.3	83.4 ± 9.7	84.9 ± 9.7	0.711^‡^	—
Pain score, median (IQR)	1 (1–1)	1 (1–1)	1 (1–1)	0.017^§^	GC > GE

^†^Data are reported as *n* (%), with *p*-values derived from Pearson’s chi-square test.

^‡^Data are reported as mean ± SD, with *p*-values determined by one-way analysis of variance (ANOVA).

^§^Data are reported as median (interquartile range, IQR), with *p*-values calculated using the Kruskal–Wallis test.

Post hoc analyses were performed only if the overall *p*-value was < 0.05.

DS, desflurance–0.9% saline group; DC, desflurance–ciprofol group; DE, desflurance–esketamine group.

## 4 Discussion

This study evaluated three sedation regimens regarding postoperative cognitive function and adverse events in 204 patients undergoing endovascular aneurysm coiling. Consistent with our results, cognitive recovery was comparable across the DS, DC, and DE groups, whereas complication rates differed significantly, supporting personalized anesthetic strategies for this population.

Preserving perioperative cognitive function is a central goal of anesthetic management, as it directly correlates with postoperative quality of life—particularly in patients with intracranial pathologies. We used the Montreal Cognitive Assessment (MoCA), a validated and sensitive instrument for perioperative cognitive monitoring (3). The repeated-measures ANOVA showed a significant main effect of time on MoCA scores (*p* < 0.001), with scores on POD 1 and POD 5 significantly higher than baseline. This improvement may reflect reduced surgical stress, the resolution of cerebral edema, or practice effects from repeated testing, and suggests that none of the regimens impaired early cognitive recovery.

Our finding of progressive postoperative improvement in MoCA scores is consistent with previous studies. Kalin et al. (22) reported similar increases at 72 h, 14 days, and 3 months after endovascular coiling. Despite different time points, the consistent pattern supports the typical cognitive recovery trajectory in these patients.

The surgical approach strongly influences cognitive outcomes. Mahajan et al. (18) found no significant cognitive differences between propofol and desflurane in craniotomy patients, with both groups showing scores below baseline. In contrast, our patients undergoing endovascular surgery achieved higher-than-baseline MoCA scores early postoperatively. This difference supports the milder cerebral injury associated with endovascular surgery over open surgery, leading to better neurocognitive recovery (4, 23, 24). Thus, anesthetic efficacy should be evaluated within the context of the surgical modality.

Our results demonstrated that desflurane combined with esketamine (DE) or ciprofol (DC) did not improve MoCA scores compared with desflurane alone (DS). The age-stratified analysis confirmed that age did not affect intergroup comparisons. Clinically, all three regimens provided equivalent neurocognitive protection, and combination regimens offered no cognitive advantage over desflurane alone.

Functional outcomes after subarachnoid hemorrhage (SAH) are influenced by hemorrhage severity (25), aneurysm location (26), complications, and pre-existing comorbidities (27). In addition, hemodynamic stability (28), rational fluid management (28, 29), and glucose control (8, 30) are critical for cognitive recovery. However, current anesthetic protocols for endovascular coiling are often extrapolated from craniotomy standards (3), leaving room for optimization specifically tailored to endovascular coiling procedures. Differences among desflurane (31), esketamine (32, 33), and ciprofol (34–36) further support the need for individualized anesthetic selection. In the present study, none of the three regimens adversely affected early cognitive function, confirming their clinical safety. In addition, while dexmedetomidine is widely recognized to attenuate POCD and delirium (37, 38), accumulating evidence indicates heterogeneous effects of dexmedetomidine on POCD, associated with variations in administration regimens, study populations, and surgical subtypes (37, 39). Consequently, whether the 0.4 μg/kg dose used in this study masked the three anesthesia regimens’ effects on early postoperative cognitive function constitutes an important confounding factor, potentially accounting for the lack of statistically significant cognitive outcome differences among the three groups.

The core of Enhanced Recovery After Surgery (ERAS) is to reduce postoperative complications. Our results revealed distinct complication profiles that support tailored anesthetic selection. Despite the routine administration of palonosetron and betamethasone for the prevention of postoperative nausea and vomiting (PONV) in adherence to established clinical practice guidelines, the incidence of postoperative nausea differed significantly (*p* < 0.001): 44.9% in the DS group, 17.9% in the DE group, and 10.3% in the DC group. The DC group also required the least metoclopramide (4.4%, *p* = 0.012), consistent with evidence that ciprofol reduces PONV risk (36). Although esketamine alone has limited antiemetic effects (40), the lower nausea rate in the DE group may reflect synergistic reductions in remifentanil and desflurane doses (40–42). For patients at high risk of PONV, in addition to combined PONV prevention regimens (20, 21), the use of desflurane monotherapy should be avoided as much as possible in clinical practice.

The dizziness incidence was lowest in the DC group (8.8%), followed by the DE (29.9%) and DS (34.8%) groups. The DE group provided the lowest pain scores (*p* = 0.012). Thus, the DC regimen is preferable for patients sensitive to dizziness, while the DE regimen is more suitable for those requiring superior analgesia.

This study has several limitations that should be considered when interpreting the findings. First, the sample size was calculated for interaction effects; larger studies are needed to confirm generalizability. Second, the MoCA alone may not detect subtle changes in specific cognitive domains; a multi-scale assessment is recommended. Third, accurate delayed cerebral ischemia (DCI) data could not be obtained, which may have influenced result interpretation despite efforts to balance baseline characteristics (Supplementary 1). Fourth, no long-term follow-up was performed. Future studies should evaluate cognitive function and quality of life at 3–6 months postoperatively to evaluate the anesthetic regimens’ sustained effects. Fifth, we also note that although a consistent dexmedetomidine administration protocol was adopted in this study, it may still be a confounding factor affecting the study outcomes.

## 5 Conclusion

In patients undergoing endovascular coiling for intracranial aneurysms, desflurane-based anesthesia supplemented with either ciprofol or esketamine demonstrates equivalent early neurocognitive recovery compared with desflurane alone. The addition of ciprofol effectively reduces postoperative nausea, vomiting, and dizziness, whereas esketamine provides superior postoperative analgesia. These findings support the use of personalized, symptom-targeted anesthetic regimens to improve perioperative safety and the quality of recovery in this high-risk patient population.

## Data Availability

The original contributions presented in the study are included in the article/supplementary material, further inquiries can be directed to the corresponding author/s.

## Footnotes

^1^https://www.ambu.com/airway-management-and-anaesthesia/laryngeal-masks/product/ambu-auraonce-disposable-laryngeal-mask.
